# Inter-individual Variability for High Fat Diet Consumption in Inbred C57BL/6 Mice

**DOI:** 10.3389/fnut.2019.00067

**Published:** 2019-05-09

**Authors:** Pablo N. De Francesco, María P. Cornejo, Franco Barrile, Guadalupe García Romero, Spring Valdivia, María F. Andreoli, Mario Perello

**Affiliations:** ^1^Laboratory of Neurophysiology, Multidisciplinary Institute of Cell Biology [Argentine Research Council (CONICET), Scientific Research Commission, Province of Buenos Aires (CIC-PBA) and National University of La Plata (UNLP)], La Plata, Argentina; ^2^Laboratory of Experimental Neurodevelopment, Institute of Development and Pediatric Research (IDIP), La Plata Children's Hospital and Scientific Research Commission, Province of Buenos Aires (CIC-PBA), La Plata, Argentina

**Keywords:** food intake, inter-individuality, palatable foods, eating behaviors, obesity

## Abstract

Since inbred C57BL/6 mice are known to show inter-individual phenotypic variability for some traits, we tested the hypothesis that inbred C57BL/6 mice display a different tendency to consume a high fat (HF) diet. For this purpose, we used a compilation of HF intake data from an experimental protocol in which satiated mice were exposed to a HF pellet every morning for 2-h over 4 consecutive days. We found that mice displayed a large degree of variability in HF intake. Since day 1 HF intake significantly correlated with HF intake in successive days, we applied a hierarchical clustering algorithm on HF intake measurements in days 2, 3, and 4 in order to classify mice into “low” or “high” HF intake groups. “Low” HF intake group showed a day 1 HF intake similar to that seen in mice exposed to regular chow, while “high” HF intake group showed a higher day 1 HF intake as compared to “low” HF intake group. Both groups of mice increased HF consumption over the successive days, but “high” HF intake group always displayed a higher HF consumption than the “low” HF intake group. As compared to “low” HF intake group, “high” HF intake group showed a higher number of dopamine neurons positive for c-Fos in the VTA after the last event of HF intake. Thus, inbred C57BL/6 mice show inter-individual variability for HF intake and such feature may be linked to a different response to the rewarding properties of the HF diet.

## Introduction

The worldwide obesity epidemic is recognized as one of the most serious global health problems. A key factor favoring body weight gain in humans is the modern lifestyle that constantly promotes the consumption of energy-dense foods. Thus, understanding the neurobiological systems that control the intake of some types of foods is essential for the development of novel strategies to tackle this growing problem. Most basic research concerning the neurobiological mechanisms controlling eating behaviors is performed in mice, which can be fed with a high-fat (HF) diet as a maneuver to mimic the increasing availability of palatable energy-dense foods in modern societies. Mice exposed to a HF diet increase their food intake, presumably because the rewarding aspects of eating override the homeostatic control of appetite and lead to the overconsumption of palatable stimuli independently of energy needs (1). Processing of rewarding aspects of food intake involve the mesolimbic pathway that includes the dopamine neurons of the ventral tegmental area (VTA), which mainly innervate the nucleus accumbens and olfactory tubercle as well as other brain areas (2). Despite many important advances in the recent years, the mechanisms by which VTA dopamine neurons modulate behavioral functions related to food reward remain poorly known (3).

In order to investigate the neurobiology of HF intake, our group set up a simple experimental paradigm in which mice display a robust intake of HF diet. In particular, satiated mice are exposed to a HF pellet every morning for 2-h over 4 consecutive days, while maintaining free access to regular chow (RC). Given the experimental conditions, the rewarding aspects of appetite play a major role regulating HF intake. In line with this notion, HF intake under this experimental paradigm increases the expression of the marker of neuronal activation c-Fos in most centers of the mesolimbic pathway (4). After collecting food intake data from mice with daily and time-limited access to HF diet over several studies (4–6), it became evident that individual mice differed notably in their tendency to consume HF diet. The presence of such phenotypic variability was masked when data was averaged, and, as a consequence, this phenomenon was overlooked in our previous publications. Here, we present a compilation of HF intake data of inbred C57BL/6 wild type mice collected in our laboratory over the past years and present a more compelling analysis. In addition, we analyzed the potential association between the individual tendency of mice to consume a HF diet and the level of activation of the VTA dopamine neurons, as estimated by the induction of c-Fos expression.

## Methods

### Animals

All studies were performed using naïve adult (2–6-month-old) mice from the inbred C57BL/6 strain that were generated in the animal facility of the IMBICE. Studies were performed using male mice in order to minimize the additional variability on eating behavior introduced by hormonal changes across ovulatory cycle. At weaning (21-day-old), mice were group-housed (6 mice/cage) and *ad libitum* fed with RC. Mice were housed under a 12-h light-dark cycle (lights switched on at 6:00 a.m.), at 22 ± 1°C room temperature. The study was carried out in accordance with the recommendations in the Guide for the Care and Use of Laboratory Animals of the National Institutes of Health. All experimentation received approval from the Institutional Animal Care and Use Committee of the Multidisciplinary Institute of Cell Biology (approval IDs 10-0113 and 15-0122).

### Diets

Diets were provided by Gepsa (Grupo Pilar, www.gepsa.com) and their color, texture and overall appearance were similar (7). Table 1 shows the composition of RC and HF diet.

**Table 1:** Composition of the diets.

	**RC**	**HF diet**
Nutrient composition (g/kg)
Protein	253	227
Total fat	36	210
Carbohydrate	500	400
Crude fiber	60	47
Ash	80	56
Moisture	71	60
Metabolizable energy (kcal/kg)	3,003	3,960

*Ingredients: Corn, gluten meal, wheat middlings, soy flour, fish meal, meat meal, wheat, barley, ground oats, alfalfa meal, sunflower flour, chicken oil, vitamin mix, mineral mix. RC and HF diet contain 36 and 210 g/kg, respectively, of chicken oil added as a source of fat*.

### Experimental Protocol

Mice were single-housed in clean cages 3 days before the experiment and *ad libitum* fed with RC. For the experiments, mice were randomly assigned into: 1) a “HF group,” which included mice daily exposed to a HF pellet inside their home cages from 9:00 to 11:00 a.m. during 4 consecutive days, or 2) a “RC group,” which included mice daily exposed to a RC pellet in their home cages from 9:00 to 11:00 a.m. All mice remained with free access to RC in the hopper. RC and HF pellets offered to mice were pre-weighed and food intake was calculated by weighing all remnants of the pellet at 11:00 a.m. and subtracting it to the initial weight. Energy intake was calculated by multiplying food intake by the respective energy content of each diet. The fourth day after the experimental protocol, a set of mice from HF and RC groups (*n* = 12 and 7, respectively) was euthanized and perfused as previously described (8); their brains were extracted, frozen, coronally cut in four equivalent series in a cryostat and stored in cryopreservant solution at −20°C until processing.

### Immunostaining

One series of brain sections was used for c-Fos and tyrosine hydroxylase (TH) immunostaining, which was performed as described before (4). Briefly, sections were treated with 0.5% H_2_O_2_ and then treated with blocking solution (3% normal donkey serum and 0.25% Triton X-100). Next, sections were incubated with an anti-c-Fos antibody (Santa Cruz Biotechnology, cat# sc-7202, 1:2,000) for 48 h at 4°C. Then, sections were incubated with a biotinylated donkey anti-rabbit antibody (Vector Laboratories, cat# BA-1000, 1:1,000) and with reagents of the Vectastain Elite ABC kit (Vector Laboratories, cat# PK-6200), according to the manufacturer's protocols. Next, sections were incubated with 3-3′-diaminobencidine (DAB)/nickel solution in order to generate a black precipitate in c-Fos positive (c-Fos+) cells. Afterwards, sections were incubated with a rabbit anti-TH antibody (Santa Cruz, cat# sc-14007, 1:20,000) for 48 h at 4°C and sequentially incubated with a biotinylated donkey anti-rabbit antibody, reagents of the Vectastain Elite ABC kit and a DAB solution without nickel in order to generate a brown precipitate in the cytoplasm of TH positive (TH+) cells. Finally, sections were mounted and coverslipped with mounting media.

### Quantitative Neuroanatomical Analysis

Blind quantitative analysis of double c-Fos+/TH+ cells was manually performed by two independent observers. Quantitative analysis was performed in sections between bregma −3.28 and −3.92 mm for the VTA. Data were expressed as the total number of c-Fos+/TH+ cells in the VTA and obtained by multiplying by four the total number of counted cells. Data were corrected for double counting, according to the method of Abercrombie (Abercrombie, 1946) where the ratio of the actual number of neurons or cell nuclei to the observed number is represented by T/(T + h) where T = section thickness, and h = the mean diameter of the neuron or cell nuclei along the z-axis. The mean diameter of the neurons was determined using Fiji. Bright-field images were acquired with 10X/0.30 and 60X/0.80 objectives using a Nikon Eclipse 50i and a DS-Ri1 Nikon digital camera with a 0.45X adapter.

### Data Processing and Statistical Analyses

Analyses were performed with a retrospective dataset coming from a combined population of 107 mice, which were tested in three independent studies following identical procedures. Part of the data has been already published (7), while the rest remained unpublished. HF group included 84 mice (36, 29, and 19 mice from studies 1, 2, and 3, respectively) and RC group included 23 mice (7, 8, and 8 mice from studies 1, 2, and 3, respectively). For each mouse, compiled information included previously recorded data (e.g., sire, mother, date of birth, litter size) as well as data of the experimental days (e.g., overnight RC intake, HF intake on successive days, body weight, date of experiment). Since no significant differences were found among studies 1, 2, and 3 for day 1 HF intake (*p* = 0.0966, Kruskal-Wallis test) or for any other analyzed parameter, all analyses were performed with data of the combined population of mice. An initial exploratory phase included the computation of a correlation matrix between variables in order to identify combinations of variables that could account for the variability of day 1 HF intake. Since day 1 HF intake positively correlated with HF intake on days 2, 3, and 4, this set of three variables was subjected to a clustering algorithm to generate a net 2-group partitioning of day 1 HF intake distribution. The classification method used was an agglomerative hierarchical clustering algorithm using a Euclidean metric for complete linkage distance. Clustering algorithms as well as the further analysis of the resulting partition were performed in KNIME (9). Statistical analysis was performed using Graphpad Prism (GraphPad Software) and differences were considered significant when *p* < 0.05. The specific statistical test for each analysis is described in the Results section. Data are presented as mean ± SEM, unless otherwise stated.

## Results

**Figure 1:**
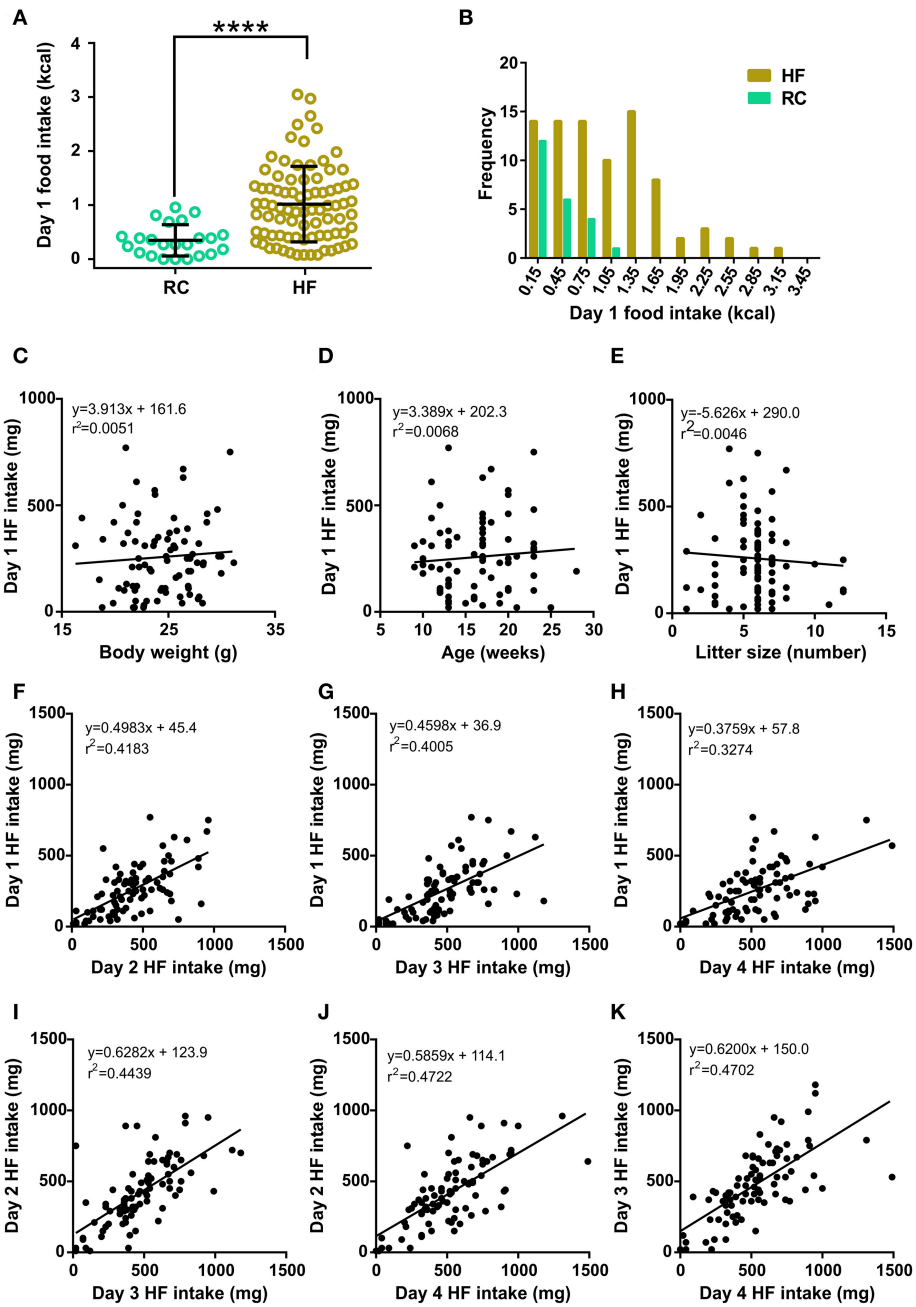
**(A)** Dot plot of the individual food intake values of RC (light green circles, *n* = 23) and HF (khaki circles, *n* = 84) groups. Lines represent the mean and SD of each group. ^****^p < 0.0001, Mann-Whitney test (*U* = 346.5). **(B)** Corresponding histograms of food intake for HF and RC mice. **(C–E)** Scatter plots of day 1 HF intake vs. body weight **(C)**, age **(D)**, and litter size **(E)**. In each case, the linear regression between both parameters is shown, along with the corresponding equation and R squared. **(F–K)** Scatter plots of day 1 HF intake vs. day 2 HF intake **(F)**, day 3 HF intake **(G)**, and day 4 HF intake **(H)**; day 2 HF intake vs. day 3 HF intake **(I)** and day 4 HF intake **(J)**, and day 3 HF intake vs. day 4 HF intake **(K)**. In each case, the linear regression between both parameters is shown with the corresponding equation and R squared. All linear regressions presented a slope significantly different from zero according to an *F* test [in **(F)**: *p* < 0.0001, *F* = 58.96; in **(G)**: *p* < 0.0001, *F* = 54.78; in **(H)**: *p* < 0.0001, *F* = 39.91; in **(I)**: *p* < 0.0001, *F* = 65.43; in **(J)**: *p* < 0.0001, *F* = 73.37 and in **(K)**: *p* < 0.0001, *F* = 72.77].

As we reported previously (7), day 1 food intake was significantly higher in the HF group as compared to the RC group (Figure 1A). Notably, food intake displayed a normal distribution in the RC group (K2 = 2.401, *p* = 0.3011, D'Agostino & Pearson omnibus normality test). In contrast, day 1 HF intake displayed a large degree of variability, ranging from 20 to 750 mg, in the HF group, and it did not adjust to a normal distribution (K2 = 9.286, *p* = 0.0096, D'Agostino & Pearson omnibus normality test, Figure 1B). Thus, we hypothesized that some intrinsic heterogeneity of the mice population was responsible for the increased variability observed in the spontaneous HF intake. In order to identify parameters that could be associated to the differential spontaneous HF intake, we looked for correlations between all recorded data for each mouse. No significant correlations were found between day 1 HF intake and body weight (Figure 1C, *F* = 0.4221, *p* = 0.5177, *F* test), age (Figure 1D, *F* = 0.5586, *p* = 0.4569, *F* test), litter size (Figure 1E, *F* = 0.3782, *p* = 0.5403, *F* test), time of the year, nor any other analyzed parameter in the dataset (data not shown). In contrast, we found positive and significant correlations among HF intake over the different events (Figures 1F–K, see *F* and *p*-values in the figure legend). These correlations suggested that the HF intake for each mouse over the consecutive days could be informative to unmask inter-individual differences in their spontaneous tendency to eat such diet.

**Figure 2:**
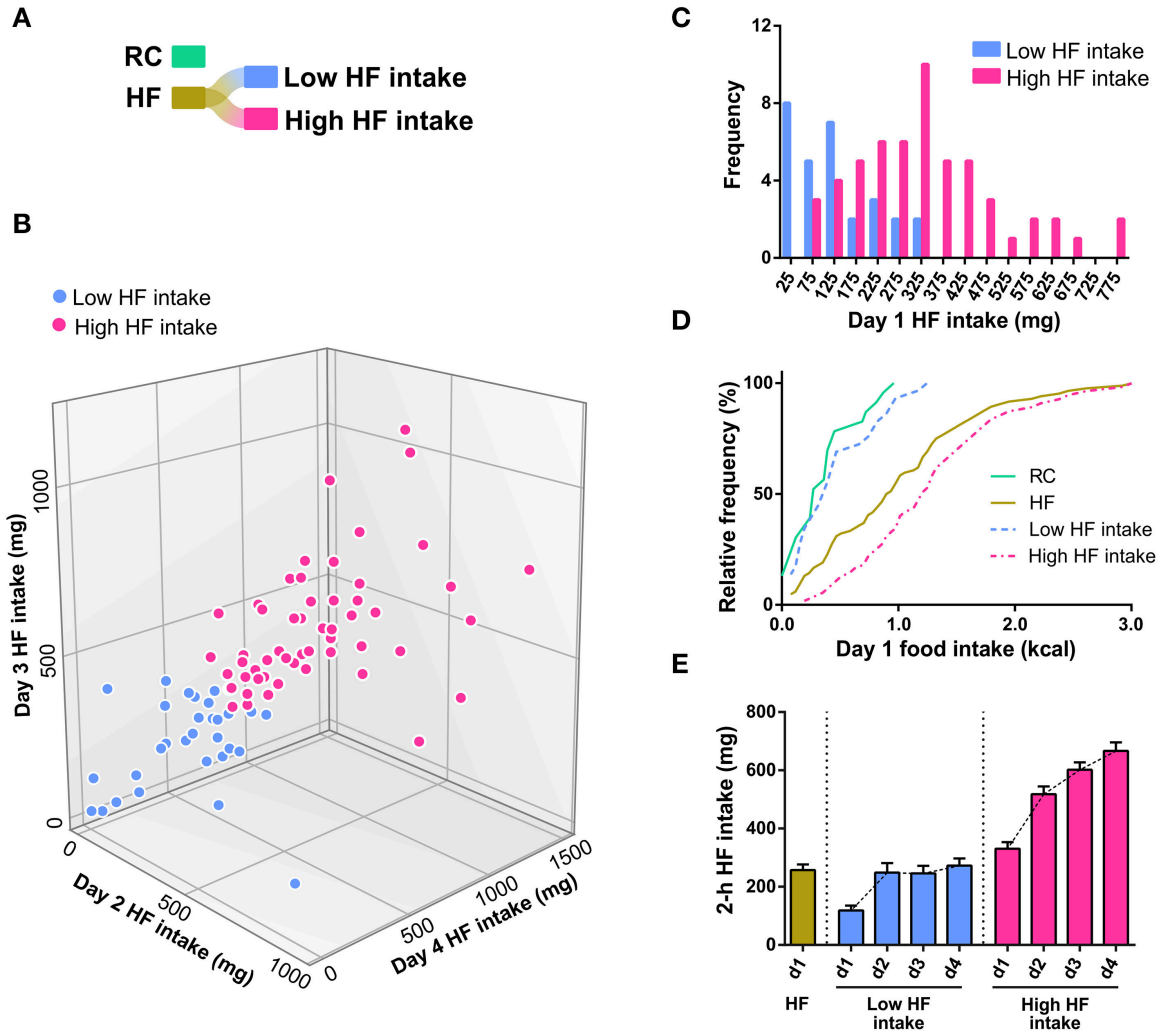
**(A)** Graphical representation of the original dataset (RC and HF), and the new partitioning of HF after clustering. The coloring scheme is maintained throughout the figures. **(B)** 3D scatter plot of day 2, day 3, and day 4 HF intake classified into the two most dissimilar sets (labeled Low HF intake and High HF intake, and colored light blue and hot pink, respectively) after applying a clustering algorithm on these same days. The classification method used was an agglomerative hierarchical clustering algorithm implemented in KNIME, using the main two clusters obtained with a Euclidean metric for complete linkage distance. Note that day 1 HF intake was explicitly excluded from this analysis. **(C)** Histograms of the day 1 HF intake from the data shown in Figure 1 **(B)**, which has been disaggregated into two clusters of maximal dissimilarity (low HF intake and high HF intake) based on the HFD intake of the following 3 days. **(D)** Cumulative distributions of day 1 food intake of the RC, HF, Low HF intake and High HF intake groups. **(E)** Bar graph showing the day 1 HF intake in the HF group and HF intake observed over successive days in the Low HF intake and High HF intake groups.

Since day 1 HF intake did not adjust to a normal distribution, we hypothesized that the HF group contained at least two subgroups of mice with different tendencies to eat HF diet. As a maneuver to generate a net 2-group partitioning of day 1 HF intake distribution, we applied a bottom-up (i.e., agglomerative) hierarchical clustering algorithm to the HF intake of days 2, 3, and 4. Note that day 1 HF intake was explicitly excluded from this analysis. Specifically, each mouse was represented as a vector containing the corresponding values of HF intake in days 2, 3, and 4. The algorithm starts with each mouse as a single cluster and tries to combine the most similar clusters into bigger clusters until all mice are combined into two distinct groups. The similarity between clusters was determined using the complete linkage method, which defines the distance between two clusters as the maximal distance between any two members within each cluster, with the Euclidean metric as a distance measure. The application of the clustering algorithm on the HF intake in days 2, 3, and 4 allowed for a net 2-group partitioning of the HF group, in which mice can be classified into “low” or “high” HF intake groups (Figures 2A,B). Notably, day 1 HF intake of “low” and “high” HF intake groups appeared normally distributed (K2 = 3.606, *p* = 0.1648 and K2 = 4.881, *p* = 0.0871, respectively, D'Agostino & Pearson omnibus normality test, Figure 2C). “Low” HF intake group represented 34.5% of total HF diet-exposed mice and was similar to the RC group in terms of both day 1 food intake (0.46 ± 0.07 vs. 0.35 ± 0.06 kcal, *p* = 0.216, *t*-test) and the cumulative distributions of day 1 food intake (Figure 2D). “High” HF intake group showed a significantly higher day 1 HF intake (1.29 ± 0.09 kcal) as compared to both “low” HF intake and RC groups (*p* < 0.0001, Kruskal-Wallis test; *p* < 0.0001, Dunn's multiple comparisons test). Notably, both “low” and “high” HF intake groups escalated HF consumption over the successive days; however, “high” HF intake group displayed a significantly larger HF consumption than the “low” HF intake group over the successive days (Figure 2E). Importantly, “low” and “high” HF intake groups contained mice from studies 1, 2, and 3, and day 1 HF intake for each group did not differ among studies [Two-way ANOVA, group vs. study: interaction *F*_(2, 78)_ = 0.3175, *p* = 0.7289; study effect: *F*_(2, 78)_ = 0.1776, *p* = 0.8376; group effect: *F*_(1, 78)_ = 28.84, *p* < 0.0001. Between-study Post-test, *p*-value adjusted for multiple comparisons: *p* = 0.9907, 0.9980, and 0.9997 for “low” HF intake group, *p* = 0.5066, 0.9277, and 0.7621 for “high” HF intake group].

**Figure 3:**
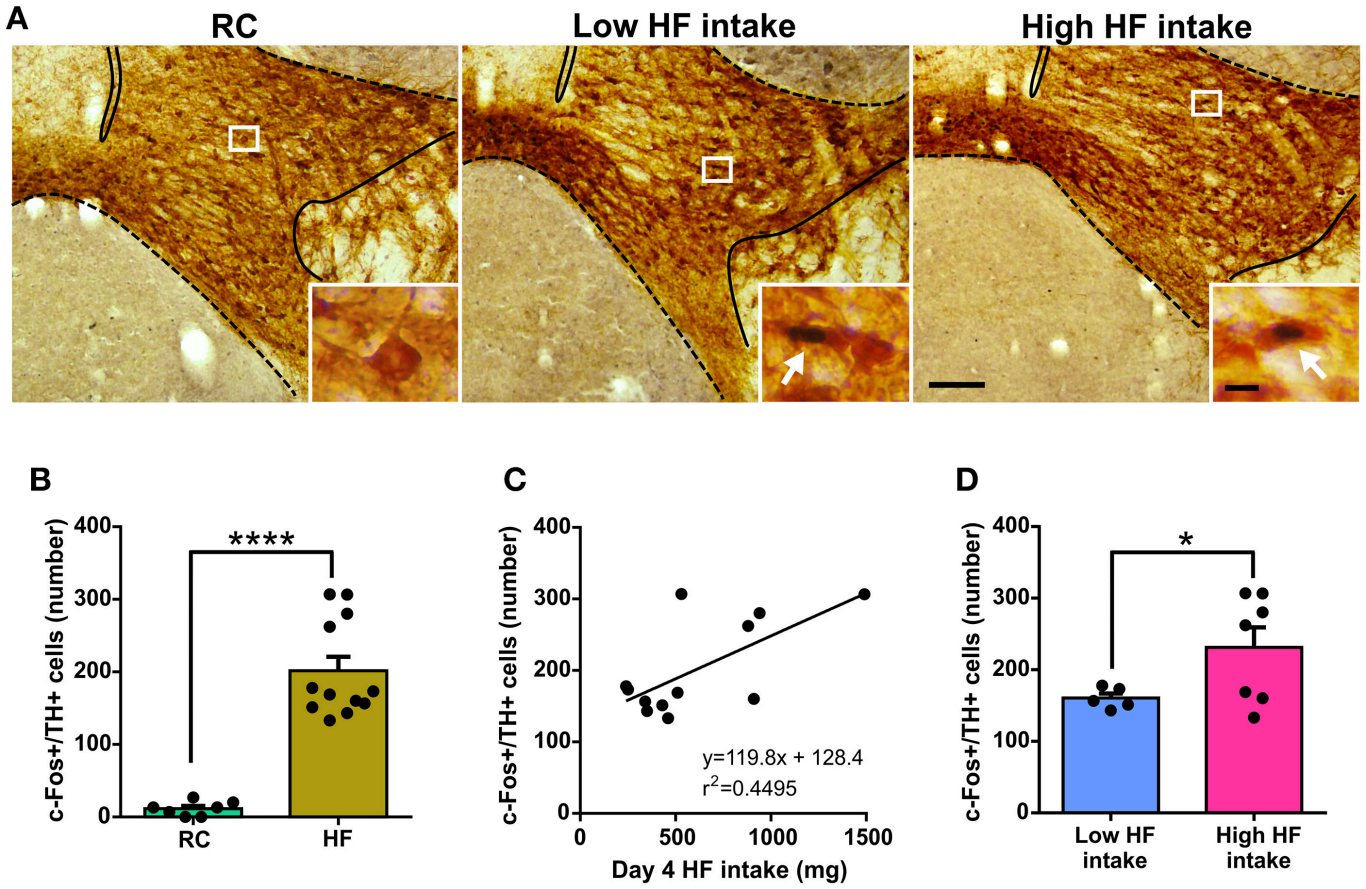
**(A)** Representative photomicrographs of the double c-Fos/TH immunostaining in the VTA of RC, Low HF intake and High HF intake groups. In each picture, the limits of the VTA are overlaid according to the delineation described in the Mouse Brain Atlas (10). Insets show, in high magnification, the areas delimited by rectangles in low magnification images. Arrows point at dual-labeled cells. Scale bars: 100 μm, low magnification and 10 μm, high magnification. **(B)** Quantitative analysis of the number of c-Fos+/TH+ cells in the VTA of RC and HF groups. Bars represent the mean ± SEM and dots represent the individual c-Fos+/TH+ values for each mouse. ^****^p < 0.0001, unpaired *t*-test with Welch's correction (*t* = 9.716, *df* = 11.83). **(C)** Scatter plot of c-Fos+/TH+ cells number vs. day 4 HF intake. The linear regression between both parameters is shown with the corresponding equation and R squared. The linear regression presented a slope significantly different from zero according to an *F* test (*p* = 0.0170, *F* = 8.166). **(D)** Quantitative analysis of the number of c-Fos+/TH+ cells in the VTA of low HF intake and high HF intake groups. Bars represent the mean ± SEM and dots represent the individual c-Fos+/TH+ values for each mouse. ^*^p < 0.05, unpaired *t*-test with Welch's correction (*t* = 2.446, *df* = 6.639).

In order to gain some insights about the neurobiological basis behind the low or high tendency of C57BL/6 mice to eat HF diet, we quantified the number of VTA dopamine neurons positive for c-Fos after food intake in the last experimental day. As previously shown (4), we confirmed that the number of c-Fos+/TH+ cells of the VTA was significantly higher in HF group as compared to the RC group (Figures 3A,B). In the HF group, the number of c-Fos+/TH+ of the VTA positively correlated with day 4 HF intake (*F* = 8.166, *p* = 0.0170, *F* test, Figure 3C). Interestingly, a significant difference was detected when the number of c-Fos+/TH+ cells of the VTA was compared between mice clustered as “low” or “high” HF intake groups (Figure 3D).

## Discussion

Individuality refers to the collection of divergent behavioral and physiological traits among individuals of a genetically identical population (11). Inbred C57BL/6 mice are known to display inter-individual phenotypic variability for some traits. For instance, C57BL/6 mice can be classified into “low” or “high” alcohol drinkers based on the bimodal pattern of distribution of alcohol preference (12–14). Inter-individual variability in alcohol preference lacks correlation with factors such as gender, locomotor activity, age and body weight, among others (12–16). “Low” or “high” alcohol drinkers display neurobiological differences in some mesolimbic brain areas such as changes in the expression of genes related to neurotransmission or to the control of epigenetic mechanisms (14) and differential activity of VTA dopamine neurons, suggesting that they differ in their response to the rewarding properties of alcohol (16). High and low alcohol drinkers also differ in the expression of orexin receptor genes in some mesolimbic centers (17). Inbred C57BL/6 mice also show inter-individual variability in response to stress (18). For instance, male mice subjected to chronic social defeat stress can be classified into “susceptible” or “unsusceptible,” based on their social interaction scores (19). Similarly, male mice can be classified into having “high” or “low” basal levels of anxiety based on their latency to emerge from their home cage into a non-familiar environment (20). Inbred C57BL/6 mice also show inter-individual variability in their susceptibility to develop obesity after long-term HF feeding, and, consequently, they can be classified into “high” or “low” gainers (21, 22). Low gainers are resistant to diet-induced obesity and remain sensitive to the anorectic actions of leptin (23). The inter-individual variability in diet-induced obesity is mainly due to changes in fat mass. A recent study also identified that male C57Bl/6JOla mice can be classified into “intolerant” or “tolerant” depending on the length of the transient increase of food intake that they display when switched from RC to HF diet (24). Both groups of mice show similar basal RC intake or initial body weight but “intolerant” mice gain more body weight than “tolerant” mice after several weeks on HF diet (24). Interestingly, long-term HF intake seems to accentuate preexisting traits of inbred mice since the HF diet-induced body weight gain was associated to the inter-individual variability in either fat mass, body weight or expression levels of genes linked to adipose tissue expansion before exposure to HF diet (21, 23, 25). Thus, current observation that inbred C57BL/6 mice display either a low or a high tendency to eat HF diet represents another trait in which this mouse strain shows inter-individual phenotypic variability.

HF intake in our experimental conditions is presumably driven by the palatable nature of the stimulus since it occurs in satiated mice, at a time of the day when spontaneous food intake is minimal, and while mice remain with free access to RC (1). It seems likely that HF intake is linked to fat-related organoleptic properties of the stimulus (e.g., flavor) because the fat content in the employed HF diet is increased at the expense of a reduction of the other macronutrients. Interestingly, the tested HF diet represents a mild rewarding appetitive stimulus as the fat content is moderately high. as compared to some commercial HF diets (26), and it does not contain added sucrose. Such mild nature of the tested stimulus may have helped to unmask the presence of the inter-individual variability in HF intake as it could be predicted that a highly palatable diet would have promoted strong food intake in the vast majority of mice.

As previously shown (4), we confirmed that daily and time-restricted HF intake in mice activates VTA dopamine neurons. Here, we also found that the number of activated VTA dopamine neurons of mice daily exposed to HF diet positively correlated with day 4 HF intake, and that mice clustered as “high” HF intake group displayed a higher number of activated VTA dopamine neurons as compared to numbers found in mice clustered as “low” HF intake group. Since HF intake *per se* activates VTA dopamine neurons and, in turn, activation of VTA dopamine neurons increases HF intake, the cause-effect relationship between these two findings remains uncertain. In a previous study, we found that the number of activated dopamine neurons of the parabrachial pigmented and interfascicular sub-regions of the VTA was higher after day 4 HF intake as compared to day 1 HF intake suggesting that these neurons were responsive to the amount of HF diet ingested (4). However, we also found that dopamine neurons of the interfascicular sub-region of the VTA were activated in anticipation to HF intake on day 4 suggesting that some VTA dopamine neurons may be also linked to the mechanisms driving HF intake in mice that are trained to daily receive it (4). Thus, our results indicate that some VTA dopamine neurons are involved in the HF intake under these experimental conditions, but their potential role as neurobiological substrates of the inter-individual variability requires future studies. In addition, further studies are required to evaluate the potential involvement of other mesolimbic areas as potential candidates mediating a different tendency of C57BL/6 mice to eat HF diet.

Since inbred mice are presumably isogenic, the presence of inter-individual variability of some traits is therefore attributable to factors that each mouse experiences, such as stochastic events or unique environmental circumstances (Champagne, 2013). Environmental factors impacting on the inter-individual variability are countless and could act intra-uterus (e.g., uterine position and blood supply, size of the litter, sex of the neighboring fetus), in the early postnatal period (e.g., size and sex ratio of the litter, maternal behavior, social interactions, stress) and/or in adulthood (dominance status, stress, health issues). A detailed description of these factors and its impact on inter-individual variability is well-described in specific reviews (27–31). Some of these environmental factors favor inter-individual variability via induction of epigenetic modifications of the genome (e.g., DNA methylation, histone acetylation, or microRNA modifications) that end up altering gene expression (11, 32). Notably, the tendency to consume HF diet was shown to be affected by environmental factors via epigenetic modifications. For instance, offspring from inbred C57BL/6 dams that consume HF diet during pregnancy and lactation show increased sucrose and fat preference in adulthood (33). Changes in the tendency to consume palatable foods were linked to a reduction in DNA methylation of some gene promoters of the dopamine and opioid systems that alters its long-term gene expression in the mesolimbic pathway (33).

The factor/s that determine the individual variability in the spontaneous tendency to consume HF diet in our experimental conditions is currently uncertain. Our analysis argues against some key factors, such as litter size, body weight or age, playing a major contribution to the individual tendency to display low or high HF intake. It is important to mention that the current study does not allow us inferring if such inter-individual phenotypic variability is due to intrinsic differences in feeding behavior or secondary to an inter-individual phenotypic variability of other mouse traits. For instance, differences in HF intake may be secondary to different anxiety/stress responses of mice to the experimental paradigm or secondary to variations in their natural biological rhythm (i.e., sleep/wakefulness, arousal, circadian rhythms) that affect food intake in the light period. Further studies are necessary to evaluate a causal association among one or more environmental factors and the tendency to eat specific types of foods.

It is important to mention that unmasking inter-individual phenotypic variability usually requires a high number of animals, in order to be more confident that a non-normal distribution of the population is not a consequence of a small sample size. Here, we analyzed combined data from 84 mice exposed to HF diet. The above-referred studies by Yang et al. (22), Koza et al. (21), or Enriori et al. (23) describing phenotypic variability for other traits in inbred C57BL/6 mice used 277, 107, or 178 mice, respectively. Additionally, it is interesting to point out that different criteria have been used to classify animals into different subgroups in a given population. For instance, Krishnan et al. (19) set a cutoff to divide “susceptible” and “unsusceptible” mice based on the social interaction scores of control mice, while Brenachot et al. (24) used a median-based split on 1-week cumulative HF intake to separate “intolerant” and “tolerant” mice. Since mice displaying a potentially different tendency to consume HF diet showed considerable overlap in day 1 HF intake, we chose to apply a hierarchical clustering algorithm on the HF intake in successive days in order to have a 2-group partitioning of day 1 HF intake. Such strategy allowed us to unmask the presence of a group of mice that displayed a HF intake on day 1 similar as seen in the control group, and that consistently showed a lower tendency to consume HF diet over the successive experimental days as well as a smaller activation of VTA dopamine neurons.

Despite the non-genetic neurobiological basis behind the inter-inter-individual variability for HF intake remains uncertain, it is intriguing to speculate the implications of the observations herein discussed on human physiology. Twin and adoption studies have clearly shown significant genetic influences on body mass index, food intake and intake patterns; however, evidence of heritability for food preferences is less strong (34). Notably, a study found that obese twins showed higher preference for fatty foods than the lean co-twin suggesting that the acquired preference for fatty foods is associated with obesity, independently of genetic background (35). Since intake of palatable foods (e.g., sugar-sweetened drinks) positively correlates with body mass index, fat mass and waist circumference (36), it seems clear that the implementation of a comprehensive nutrition education that improves eating habits toward the preference/acceptance of healthy foods would help against the worldwide obesity epidemic.

## Data Availability

The datasets for this manuscript are not publicly available because the article reports the analysis of internal data of the laboratory. We can make the data publicly available if needed. Requests to access the datasets should be directed to mperello@imbice.gov.ar

## Author Contributions

MC, PD, and MP contributed to the conception and design of the study. MC, PD, FB, GG, and MA organized the database. PD and MC performed the statistical analyses. SV, MC, FB, and GG obtained the experimental data. MC and MP wrote the first draft of the manuscript. MC, PD, FB, GG, MA, and MP wrote sections of the manuscript. All authors contributed to manuscript revision, read, and approved the submitted version.

### Conflict of Interest Statement

The authors declare that the research was conducted in the absence of any commercial or financial relationships that could be construed as a potential conflict of interest.
